# The importance of systemic inflammatory response measurements as pretransplant risk factors for outcome after allogeneic haematopoietic cell transplantation

**DOI:** 10.1111/bjh.70049

**Published:** 2025-07-29

**Authors:** Hartmut Bertz, Jörg Sahlmann, Donald C. McMillan, Claudia Wehr, Jesus Duque‐Afonso, Kristina Maas‐Bauer, Ralph Wäsch, Julia Nikolaychuk, Christine Greil, Jann Arends, Jürgen Finke, Robert Zeiser

**Affiliations:** ^1^ University Medical Center Department of Internal Medicine, Hematology, Oncology & Stem Cell Transplantation, Faculty of Medicine Freiburg Germany; ^2^ Faculty of Medicine, Institute of Medical Biometry and Statistics (IMBI), University Medical Center Freiburg, University of Freiburg Freiburg Germany; ^3^ Academic Unit of Surgery, School of Medicine Dentistry and Nursing, College of Medicine, Veterinary and Life Sciences, University of Glasgow Glasgow UK

**Keywords:** allogeneic haematopoietic stem cell transplantation, modified Glasgow prognostic score, outcome, risk factors, systemic inflammation response measures

## Abstract

Systemic inflammatory response (SIR) measures are known prognostic values in patients with solid tumours. Little is known about their impact in haematological diseases or allogeneic haematopoietic cell transplantation (alloHCT). Therefore, we evaluate the association of pretransplant inflammatory markers with the clinical outcome in a prospective analysis of alloHCT. We included 2201 consecutive patients undergoing their first alloHCT. At admission, C‐reactive protein (CRP), serum albumin (sALB), a composite inflammatory score (modified Glasgow prognostic score, mGPS) and the body mass index (BMI) were recorded. These, the EBMT score and the HCT‐specific comorbidity index (HCT‐CI) were statistically analysed for overall/progression‐free survival (OS/PFS), non‐relapse mortality (NRM), relapse and graft‐versus‐host disease (GvHD). CRP was increased in 34.8%, 17% had a low sALB, 2.9% an increased (≥35 kg/m^2^) and 3% a very low BMI (<18.5 kg/m^2^). For mGPS, we observed score 1 (24%) and 2 (11%). A multivariable model explored mGPS 1 (SHR 1.301) and mGPS 2 (SHR 1.915) as significant risk factors (*p* < 0.001) for OS and for PFS (mGPS 1 (SHR 1.202); mGPS 2 (SHR 1.765)). Only mGPS 2 (HR 1.671; *p* < 0.001) revealed influence on NRM. Pretransplant SIR markers and their combination (mGPS) displayed a highly significant association with clinical outcome, possibly enriching present alloHCT risk scores.

## Graphical abstract

In oncological patients SIR measures like the mGPS have great impact on prognosis at starting therapy. We recorded at admission for allo‐HCT the mGPS in 2201 patients: score 0 CRP/sALB normal; score 1 CRP >10mg/dl; score 2 CRP >10mg/dl + sALB <35g/L. Multivariate analysis revealed a significant impact with a low GPS score for OS: mGPS 1 vs. 0: SHR 1.301(CI 95% 1.14‐1.48) p<0.001; mGPS 2 vs. 0: SHR 1.915 (CI 95% 1.58‐2.31) p<0.001 and PFS: mGPS 1 vs. 0 SHR 1.202 (CI 95% 1.06‐1.36) p=0.0043, mGPS 2 vs. 0: SHR 1.765 (CI 95% 1.47‐2.12) p<0.001. SIR measures (CRP and sALB) and their combination (mGPS) display a highly significant association with clinical outcome.

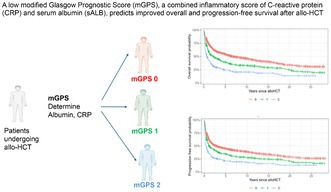

## INTRODUCTION

Several clinical prognostic pretransplantation scores have been developed for patients scheduled for alloHCT regarding their outcome, namely the haematopoietic cell transplantation specific comorbidity index (HCT‐CI)^1^ and EBMT score.^2^ They have been validated to predict overall/progression‐free survival (OS/PFS), relapse, non‐relapse mortality (NRM) and acute or chronic graft‐versus‐host disease (a/cGvHD). Furthermore, patients' nutritional status,^3^ especially malnutrition defined as low body mass index (BMI <18.5 kg/m^2^; WHO definition), are known predictors for a worse response‐ AML induction therapy^4^ and in alloHCT.^5^, ^6^ This is associated with a higher risk of complications like infections and wound healing problems in patients with oncological malignancies.^3^ Over the last decades, the systemic inflammatory response at diagnosis and during therapy has demonstrated a strong impact on outcome.^7^, ^8^ The Glasgow prognostic score (GPS)^9^ and its modified version (mGPS)^10^ are now established and validated inflammation‐based scores of prognostic significance for predicting patients' outcomes and quality of life, primarily assessed in oncological diseases.^9^, ^10^, ^11^ The mGPS has been discussed as a kind of ‘laboratory cachexia’.^12^ The on‐treatment mGPS was recently evaluated in studies involving immunotherapy for metastatic urothelial carcinoma and should be used along with imaging scans to better predict the outcome and benefit in such patients.^13^ The mGPS has revealed its quality in haematological diseases as a prognostic factor for OS/PFS in single publications on NHL,^14^ AML,^15^ Hodgkin lymphoma^16^ and multiple myeloma patients receiving an autologous HCT.^17^ The GPS/mGPS applies the combination of serum albumin (sALB), a biomarker for nutrition and the inflammation response status,^18^ and C‐reactive protein (CRP) also a marker for the inflammatory response system. So far, these inflammatory biomarkers have not been well investigated in conjunction with alloHCT. For example, a small cohort of patients with a high GPS showed a worse outcome,^19^, ^20^ low serum albumin^21^, ^22^ or a high CAR (C‐reactive protein/serum albumin ratio)—all of which worsen outcomes.^23^, ^24^ Of the established HCT scores, none includes inflammation markers or nutritional status, except BMI >35 kg/m^2^ entailing one point in the HCT‐CI.^1^ For example, the combination of clinical symptoms and biomarkers has recently been established for the outcome of aGvHD in the novel MAGIC score.^25^

Here, we report on detailed multivariable analyses in all 2201 consecutive adult patients receiving their first alloHCT at our centre regarding nutrition, systemic inflammatory response biomarkers and their combination (BMI, sALB, CRP, mGPS) and alloHCT‐specific outcomes.

## PATIENTS AND METHODS

### Patients

All 2201 consecutive patients undergoing their first alloHCT between 1 January 1996 and 31 December 2023 in the Section for Allogeneic Stem Cell Transplantation at Freiburg University Medical Center were included in our primary assessment. This is a study from our prospectively collected database of patients undergoing alloHCT. In this time period, nine further patients were admitted for alloHCT, but died during conditioning therapy and were thus excluded from analyses (Table 1; Table S1).

**TABLE 1:** Patients' characteristics by mGPS.

	*n* = 2201	mGPS 0 (*N* = 1434)	mGPS 1 (*N* = 521)	mGPS 2 (*N* = 246)
Time period	1996–2023			
Age at HCT (years)				
Mean ± SD	52.59 ± 14.29	51.92 ± 14.30	53.04 ± 14.42	55.58 ± 13.60
Min	16.3	16.3	18.8	19.3
Median (Q1, Q3)	55.0 (43.1, 64.0)	53.8 (42.1, 63.3)	56.0 (43.9, 64.8)	58.5 (48.9, 65.9)
Max	79.1	79.1	77.1	78.9
Gender	***n* (%)**	***n* (%)**	***n* (%)**	***n* (%)**
Male	1288 (59)	831 (58)	317 (61)	140 (57)
Female	913 (41)	603 (42)	204 (39)	106 (43)
Diagnosis				
AML, MDS, MPN, CML	1466 (67)	914 (64)	376 (72)	176 (72)
ALL, NHL, HL, MM, CLL	686 (31)	493 (34)	138 (26)	55 (22)
Non‐malignant	49 (2)	27 (2)	7 (1)	15 (6)
Remission at HCT				
Early (CR1/CP1)	559 (25)	464 (32)	75 (14)	20 (8)
Advanced (>CR1/CP1/RA/RARS)	1642 (75)	970 (68)	446 (86)	226 (92)
Conditioning regimens				
Myeloablative (MAC)	697 (32)	485 (34)	161 (31)	51 (21)
‘Reduced’ (RIC)	1504 (68)	949 (66)	380 (69)	195 (79)
Donors				
Related (sibling/family/twin)	721 (33)	468 (33)	176 (34)	77 (31)
Unrelated	1480 (67)	966 (67)	345 (66)	169 (69)
Female donor/male recipient	473 (21)	299 (21)	122 (23)	52 (21)
Graft source				
BM	182 (8)	118 (8)	49 (9)	15 (6)
PBSC	2018 (92)	1315 (92)	472 (91)	231 (94)
Cord‐blood	1 (0)	1 (0)		
Inflammation parameters				
Serum albumin (g/L)				
Mean ± SD		41.43 ± 5.66	40.35 ± 3.84	30.27 ± 3.17
Min	8	8	35	20
Median (Q1, Q3)	41	42.0 (38.0, 45.0)	40.0 (38.0, 43.0)	31 (28.0, 33.0)
Max	75	75	70	34
C‐reactive protein (mg/L)				
Mean ± SD		3.93 ± 2.460	42.28 ± 47.865	79.56 ± 68.335
Min	0	0	10.4	10.1
Median (Q1, Q3)	5.4	3.0 (3.0, 5.0)	25.0 (15.0, 49.0)	58.4 (26.1, 110.8)
Max	500	10	500	373
BMI (kg/m^2^)				
Mean ± SD		25.488 ± 4.37	25.342 ± 4.53	24.472 ± 4.56
Min	15.06	15.06	15.62	15.55
Median (Q1, Q3)	24.8	24.86 (22.41, 27.77)	24.90 (22.04, 28.09)	23.80 (21.20, 26.98)
Max	52.09	43.18	47.06	52.09
BMI (kg/m^2^)	***N* (%)**			
<18.5	67 (3)	38 (3)	17 (3)	12 (5)
18.5 to <25.0	1075 (49)	692 (48)	249 (48)	134 (54)
25.0 to <30.0	737 (33)	483 (34)	180 (35)	74 (30)
30.0 to <35.0	253 (12)	180 (13)	58 (11)	21 (9)
≥35.0	63 (3)	41 (3)	17 (3)	5 (2)

Abbreviations: ALL, acute lymphoid leukaemia; AML, acute myeloid leukaemia; BM, bone marrow; BMI, body mass index; CLL, Chronic Lymphoid Lymphoma; CML, chronic myeloid leukaemia; CP, chronic phase; CR, complete remission; HL, Hodgkin lymphoma; Max, maximum; MDS, myelodysplastic syndrome; Min, minimum; MM, multiple myeloma; MPN, myeloproliferative neoplasm; NHL, Non‐Hodgkin lymphoma; PBSC, peripheral blood stem cells; Q, quartile; RA, refractory anaemia; RARS, refractory anaemia with ring sideroblasts; SD, standard deviation.

The 913 female and 1288 male patients had a median age of 55.0 years (range 16.3–79.1). Diagnoses were myeloid (66.6%) and lymphoid (31.2%) malignancies and others/non‐malignant disorders (2.2%). In 1444 patients, the disease duration from diagnosis until transplantation was below 365 days. Only 559 (25%) patients had early disease at the time of alloHCT, defined as CR1/CP1.

### Conditioning regimen, GVHD prophylaxis, graft and donors

Of the 2201 patients, 92% were transplanted with peripheral blood stem cells (PBSC) and 8% with bone marrow after receiving a myeloablative conditioning regimen (MAC; 32%) or a reduced conditioning regimen (RIC 68%). One patient received cord blood as a graft.

GvHD prophylaxis consisted primarily of ciclosporin (93.4% of patients) mainly starting at day −3 at a dose of 2.5 mg/kg b.i.d. (trough level 250–350 ng/mL) with different combination partners. Standard supportive care was given as described previously.^26^ The donor was related (*n* = 706), unrelated (*n* = 1480) or a monozygotic twin (*n* = 15). Median donor age was 35 years (range 7–79); 841 patients had a donor ≥40 years old. A female individual donated for a male recipient in 473 (21%) transplantations.

All patients signed informed consent for alloHCT and prospective data collection and were treated according to the Declaration of Helsinki.

### Nutrition counselling

Every patient admitted for alloHCT receives nutrition counselling shortly after admission by the dietitians from the Section of Nutrition and Dietetics. They are advised according to specific guidelines established for patients during alloHCT, that is, so‐called allogeneic transplantation cost as described already.^26^, ^27^

### Statistical analysis

Statistical analyses were performed with R V4.4.1 (R Foundation for Statistical Computing, Vienna, Austria) and RStudio V2024.04.2 (Posit Software, PBC). OS/PFS were calculated as time from alloHCT until death from any cause/disease progression or death from any cause. In case the event of interest was not observed, times until the patient was last seen alive/alive without disease progression were regarded as censored observations. Censoring occurred at the last contact till 31 March 2024. The Kaplan–Meier method and Cox regression models were applied in our analysis of OS and PFS according to Fine and Gray. Median follow‐up was determined with the inverse Kaplan–Meier method. Relapse and death without prior relapse (non‐relapse mortality, NRM) were considered as competing events. Cumulative incidence rates are displayed and run with Fine and Gray regression models. We constructed multivariable models starting with a full model including BMI (classified), mGPS and those variables showing a univariate *p* < 0.05 and no collinearity with each other. Backward variable selection was then performed (selection criterion *p* < 0.1) to arrive at the final models presented here.

## RESULTS

### Outcome

On 31 March 2024, 876 patients (40%) were known alive and one patient lost follow‐up; the last contact was used for median follow‐up as 3085 days (range 100–10 282) or 103 months (range 3–343 months). Reasons for death were NRM (*n* = 737; 33.5%), mainly due to infections (*n* = 390; 17.7%) or relapse/PD as well in 587 patients (26.7%).

Not included in our analyses were nine patients (male/female: 5/4; median age 69 years (range 49–77)), who were admitted for transplantation but died during conditioning therapy of septic shock (*n* = 7), ARDS (*n* = 1) and myocardial infarction (*n* = 1). Their poor general condition was verified via high inflammation markers, that is, mGPS median 2 (0–2), CRP median 76 mg/L (3–150), sALB 33 g/L (26–46) and high score for the HCT‐CI with median 4 (1–7). The median BMI was 26.7 kg/m^2^ (18.6–40.2). The EBMT score was not evaluated because the score is donor dependent (Table S1).

Engraftment with WBC counts ≥1 × 10^9^/L was achieved on day +14 (median, range 1–45) in 2139/2201 patients (97%). Sixty‐two patients died before engraftment or suffered primary graft failure. Stable platelet counts ≥20 × 10^9^/L were reached by day +13 (median, range 1 to >365); 184 patients died prior to stable platelet engraftment or experienced primary graft failure.

At day +30, haematological CR was achieved in 1859 patients (85%) and PR in 118 patients (5%). *N* = 224 patients (10%) did not respond or were not evaluable for diagnostic procedures at day +30.

### Nutrition‐/inflammation‐status

Median BMI was 24.82 kg/m^2^ (range 15.06–52.09). Malnutrition (BMI <18.5 kg/m^2^ according to the WHO definition) was observed in *n* = 67 (3.0%) and overweight (BMI ≥35 kg/m^2^) in *n* = 63 (2.9%) patients. Median sALB was 41 g/L (range 8–75; normal value ≥35 g/L). According to the mGPS, the sALB was in the normal range in 83.0% (*n* = 1827) and was <35 g/L in 17.0% (*n* = 374). The median CRP was 5.4 mg/L (range 0–500; normal value <3 mg/L); according to the mGPS, CRP was in the normal range (≤10 mg/L) in 65.2% (*n* = 1434), and increased in 34.8% (*n* = 767) (Table 2).

**TABLE 2:** Relevant inflammation and risk scores for outcome.

	*N*	%
Modified GPS		
0	1434	65
1	521	24
2	246	11
HCT‐CI		
0–2	1226	56
≥3	975	44
EBMT score		
0–2	225	10
≥3	1976	90

Abbreviations: EBMT, European Society for Blood and Marrow Transplantation; GPS, Glasgow prognostic score; HCT‐CI, haematopoietic cell transplantation specific comorbidity index.

### Scores

The mGPS is defined as: score 0—normal CRP/sALB, score 1—increased CRP (>10 mg/L) and score 2—increased CRP (>10 mg/L) and low sALB (<35 g/L): *n* = 1434 patients revealed a mGPS 0 (65%), *n* = 521 patients (24%) score 1 and *n* = 246 patients (11%) score 2 respectively (Table 2).

The median HCT‐CI score was 2 (range 0–15); 1226 patients had a ≤2 score (56%); and 975 patients (44%) had a score ≥3 predicting a worse outcome.

Comparing mGPS and HCT‐CI, no correlation was statistically found. Furthermore, mGPS is evenly distributed over all HCT‐CI score groups, and mGPS 1/2 increase monotonically with increasing HCT‐CI score categories.

The median EBMT score was 5 (range 0–7); 225 patients had a score of 0–2 (10%) and 1976 patients had a score of 3–7 (90%) predicting a worse outcome.

### Overall/progression‐free survival

OS rates at 1, 2, 5 and 10 years were 69.0% (95% CI, 67.0–70.9), 58.3% (95% CI, 56.3–60.4), 47.4% (95% CI, 45.3–49.6) and 40.2% (95% CI, 38.0–42.4). For univariate regression analysis according to Fine and Gray parameters, their SHR are listed in Table S2. Cumulative death rates at 1, 2, 5 and 10 years were 0.31 (95% CI, 0.291–0.330), 0.417 (95% CI, 0.396–0.438), 0.526 (95% CI, 0504–0.547) and 0.598 (95% CI, 0.576–0.620) respectively (Table 3).

**TABLE 3:** Multivariable risk factors for outcome.

Variable	Multivariable analysis		
	SHR	95% CI	*p*‐value
Overall survival			
Remission >CR/CP vs. CR1/CP1	1.661	1.39–1.99	<0.001
>CR1/CP>1 vs. CR1/CP1	1.326	0.99–1,78	0.061
mGPS 1 vs. 0	1.301	1.14–1.48	<0.001
mGPS 2 vs. 0	1.915	1.58–2.31	<0.001
HCT‐CI 1/2 vs. 0	1.306	1.12–1.53	<0.001
HCT‐CI ≥3 vs. 0	1.428	1.23–1.66	<0.001
EBMT 3–7 vs. 0–2	1.280	0.97–1.69	0.0790
Graft PBSC vs. BM	0.973	0.76–1.25	0.83
Female donor/male recipient	1.247	1.10–1.42	<0.001
aGvHD °I vs. 0	0.822	0.70–0.96	0.013
aGvHD °II vs. 0	0.947	0.80–1.12	0.53
aGvHD °III vs. 0	1.287	1.08–1.54	0.0056
aGvHD °IV vs. 0	2.799	2.23–3.52	<0.001
Limited cGvHD vs. none	0.490	0.41–0.58	<0.001
Extensive cGvHD vs. none	0.487	0.43–0.55	<0.001
Progression‐free survival			
Remission >CR/CP vs. CR1/CP1	1.700	1.44–2.01	<0.001
CR/CP>1 vs. CR1/CP1	1.370	1.03–1.82	0.0300
Graft PBSC vs. BM	0.895	0.72–1.12	0.33
mGPS 1 vs. 0	1.202	1.06–1.36	0.0043
mGPS 2 vs. 0	1.765	1.47–2.12	<0.001
HCT‐CI 1/2 vs. 0	1.175	1.01–1.36	0.0340
HCT‐CI ≥3 vs. 0	1.245	1.08–1.44	0.0027
Female donor/male recipient	1.149	1.01–1.31	0.0340
Donor unrelated vs. related	0.878	0.78–0.99	0.0390
aGvHD °I vs. 0	0.828	0.71–0.96	0.0130
aGvHD °II vs. 0	0.905	0.77–1.06	0.23
aGvHD °III vs. 0	1.142	0.97–1.35	0.12
aGvHD °IV vs. 0	2.217	1.83–2.68	<0.001
Limited cGvHD vs. none	0.488	0.41–0.57	<0.001
Extensive cGvHD vs. none	0.457	0.40–0.52	<0.001
Relapse incidence			
Remission >CR/CP vs. CR1/CP1	1.940	1.60–2.35	<0.001
CR/CP>1 vs. CR1/CP1	1.378	0.97–1.96	0.076
mGPS 1 vs. 0	1.120	0.95–1.32	0.17
mGPS 2 vs. 0	0.997	0.78–1.27	0.98
Conditioning RIC vs. MAC	0.645	0.55–0.76	<0.001
Graft PBSC vs. BM	1.254	0.96–1.63	0.093
Donor unrelated vs. related	0.697	0.60–0.81	<0.001
aGvHD °I vs. 0	1.127	0.94–1.35	0.19
aGvHD °II vs. 0	1.018	0.83–1.25	0.87
aGvHD °III vs. 0	0.925	0.72–1.19	0.55
aGvHD °IV vs. 0	0.385	0.24–0.61	<0.001
Limited cGvHD vs. none	0.747	0.61–0.91	0.0041
Extensive cGvHD vs. none	0.421	0.35–0.51	<0.001
Non‐relapse mortality			
mGPS 1 vs. 0	1.124	0.94–1.34	0.19
mGPS 2 vs. 0	1.671	1.33–2.10	<0.001
HCT‐CI 1/2 vs. 0	1.205	0.98–1.48	0.0790
HCT‐CI ≥3 vs. 0	1.382	1.14–1.68	0.0012
Conditioning RIC vs. MAC	1.793	1.50–2.15	<0.001
Graft PBSC vs. BM	0.668	0.50–0.88	0.0049
Donor unrelated vs. related	1.286	1.09–1.52	0.0029
Female donor/male recipient	1.252	1.06–1.48	0.0081
aGvHD °I vs. 0	0.750	0.61–0.92	0.0066
aGvHD °II vs. 0	0.968	0.78–1.20	0.77
aGvHD °III vs. 0	1.231	0.97–1.56	0.0850
aGvHD °IV vs. 0	3.763	2.89–4.89	<0.001
Limited cGvHD vs. none	0.577	0.45–0.74	<0.001
Extensive cGvHD vs. none	0.992	0.84–1.17	0.92

Abbreviations: aGvHD, acute graft‐versus‐host disease; BM, bone marrow; cGvHD, chronic graft‐versus‐host disease; CP, chronic phase; CR, complete remission; EBMT, European Society for Blood and Marrow Transplantation; HCT‐CI, haematopoietic cell transplantation‐specific comorbidity index; MAC, myeloablative conditioning; mGPS, modified Glasgow prognostic score; PBSC, peripheral blood stem cells; RIC, reduced intensity conditioning.

**FIGURE 1:**
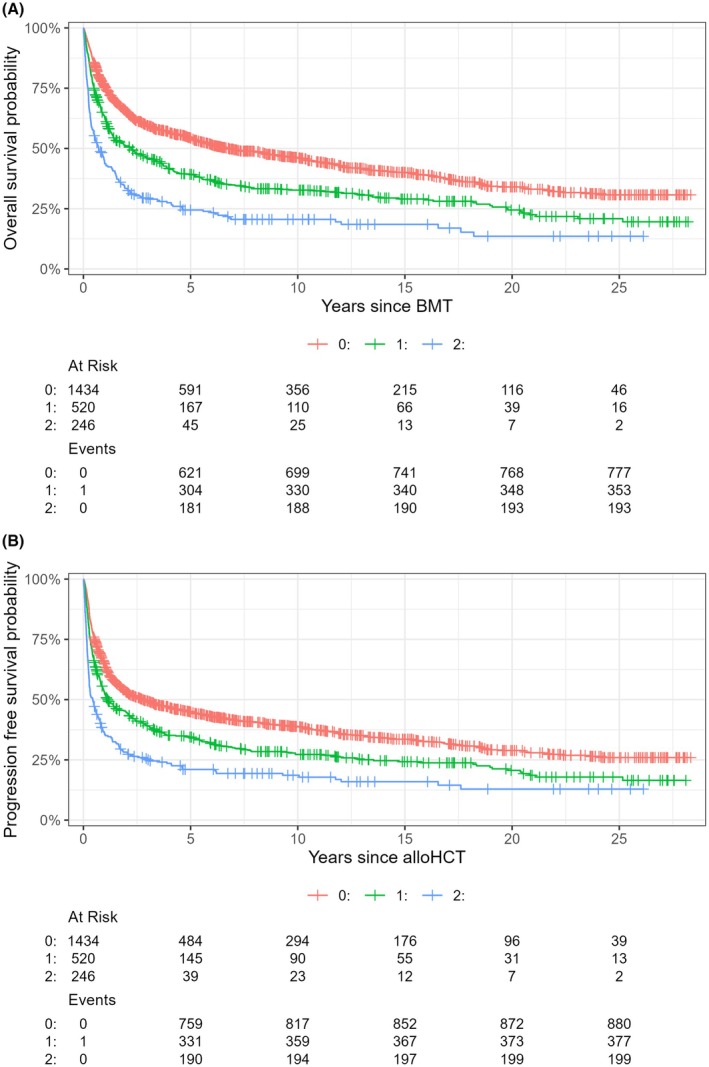
Kaplan–Meier curves showing the relationship between the different modified Glasgow prognostic scores (mGPS) regarding (A) overall survival and (B) progression‐free survival probability.

**FIGURE 2:**
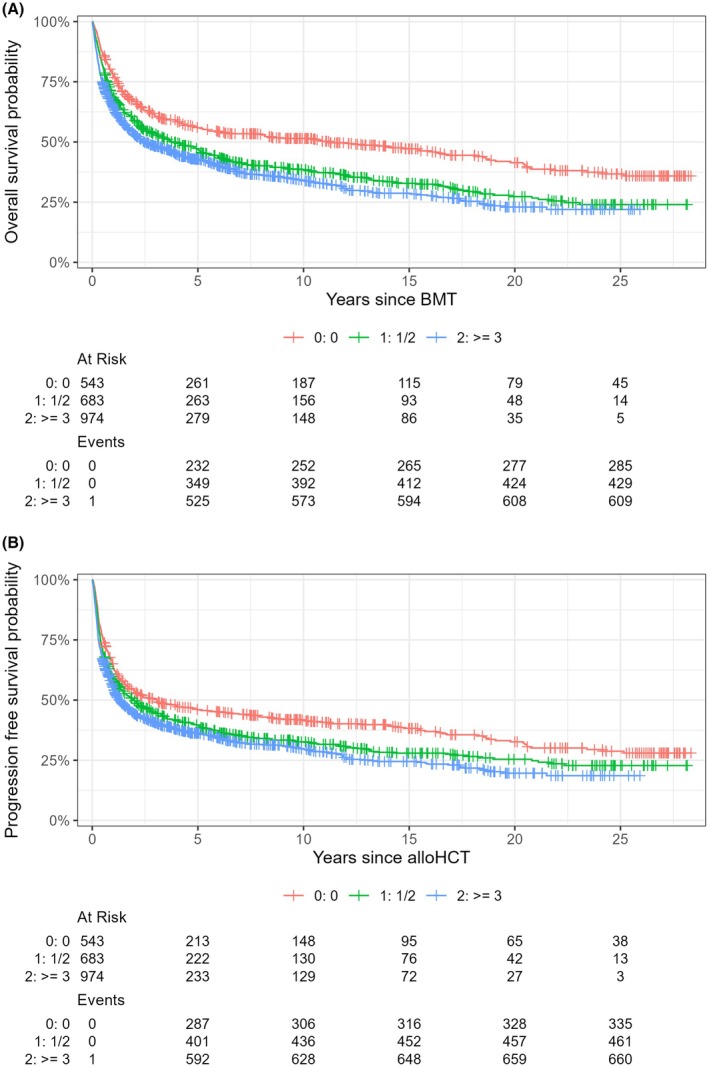
Kaplan–Meier curves showing the relationship between the different HCT‐CI risk scores regarding (A) overall survival and (B) progression‐free survival probability. HCT‐CI, haematopoietic cell transplantation‐specific comorbidity index.

**FIGURE 3:**
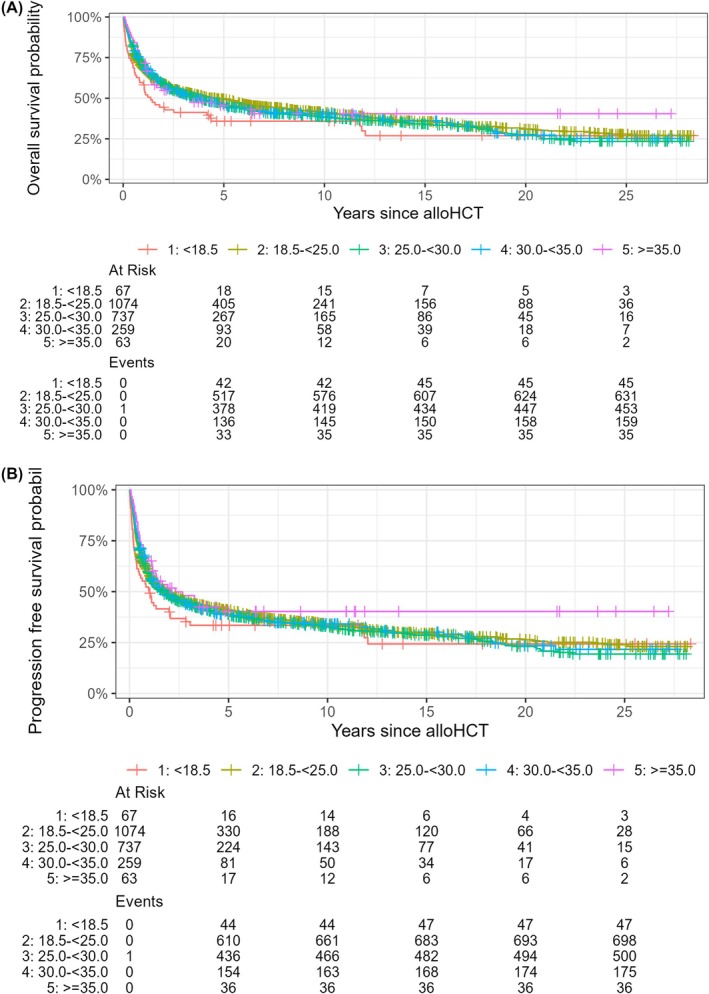
Kaplan–Meier curves showing the relationship between the different BMI categories regarding (A) overall survival and (B) progression‐free survival probability. BMI, body mass index.

Multivariable analysis revealed regarding the markers and scores of special interests the most negative influencing factors: mGPS 1 (SHR 1.301 [95% CI 1.14–1.48]; *p* < 0.001), mGPS 2 (SHR 1.915 [95% CI 1.58–2.31]; *p* < 0.001); HCT‐CI ≥3 (SHR 1.428 [95% CI 1.23–1.664]; *p* < 0.001) and HCT‐CI 1/2 (SHR 1.306 [95% CI 1.12–1.53]; *p* < 0.001). (Figures 1, 2, 3; Figures S1, S2A and S3A) (for further results, see Table 3; Table S5).

PFS at 1, 2, 5 and 10 years was 57.9% (95% CI, 55.9–60.1), 48.3% (95% CI, 46.3–50.5), 39.8% (95% CI, 37.7–41.9) and 33.8% (95% CI, 31.7–36.0) respectively. Again, for univariate regression analysis, see Table S2. In multivariable analysis of markers and scores, the most strongly influencing factors were mGPS 2 (SHR 1.765 [95% CI 1.47–2.12]; *p* < 0.001) and HCT‐CI 1/2 (SHR 1.175 [95% CI 1.01–1.36]; *p* = 0.0340) (Figures 1, 2, 3; Figures S1, S2B and S3B; Table 3; Table S5).

### Non‐relapse mortality/relapse

NRM cumulative incidence rates at 1, 2, 5 and 10 years were 16.3% (95% CI, 14.9–17.9), 20.4% (95% CI, 18.7–22.0), 25.3% (95% CI, 23.5–27.1) and 29.9% (95% CI, 28.0–31.9) (Figure S4). Again, for univariate regression analysis, see Table S2. Multivariable analysis revealed the following markers and scores as risk factors highly significant for NRM: mGPS score 2 (SHR 1.671 [95% CI 1.33–2.10]; *p* < 0.001) and HCT‐CI score ≥3 (SHR 1.382 [95% CI 1.14–1.68]; *p* = 0.0012) (Table 3).

As mentioned above, infections were the cause of death in 390 patients. Looking at the three scores, we note that patients with an mGPS 2 or a higher HCT‐CI score (≥3) are at risk of dying earlier of infections. In patients with mGPS 2, death due to infection was on median day +58.5 (range 3–2516), with mGPS 1 on median day +160 (range 0–2802) and mGPS 0 on median day +238 (range 1–6695). Death of infection with HCT‐CI 0 was on median day +294 (range 15–3105), HCT‐CI 1/2 on median day +229 (range 5–6103) and HCT‐CI ≥3 on median day +119 (range 0–6695). Considering the various EBMT scores, we observe that the differences are not particularly striking—score 0/1: death on median day +433 (range 162–1927), score 2: on median day +318 (range 15–997), score 3 on median day +244.5 (range 5–3801) and scores ≥4 on median day +150.5 (range 0–6695) (Table S3).

For relapse, the cumulative incidence rates at 1, 2, 5 and 10 years were 24.2% (95% CI, 22.5–26.0), 30.2% (95% CI, 28.2–32.1), 35.1% (95% CI, 33.1–37.1) and 37.5% (95% CI, 35.4–39.5) respectively (Figure S4). Again, univariate regression analysis is listed in Table S3. No inflammation marker/score had in the multivariable analysis a significant influence on the relapse incidence (further results see Table S3).

### Graft‐versus‐host disease (Table S4)

Clinically relevant aGvHD °II–IV developed in 699 patients (31.8%), and °III–IV in 373 patients (17.0%), with cumulative incidences at day +100 of 30.4% (95% CI, 28.4–32.4) and 16.3% (95% CI, 14.8–17.9), and at day +365 of 33.2% (95% CI, 31.2–35.3) and 17.8% (95% CI: 16.2–19.4) respectively. For univariate regression analysis, see Table S4.

According to multivariable analysis, significant risk factors of the markers and scores for °II–°IV aGvHD were BMI 30.0 to <35.0 kg/m^2^ (SHR 1.469 [95% CI, 1.10–1.95]; *p* = 0.0081) and for °III–°IV aGvHD as well as BMI 30.0 to <35.0 kg/m^2^ (SHR 1.756 [95% CI, 1.26–2.44]; *p <* 0.001) and HCT‐CI ≥3 (SHR 1.543 [95% CI, 1.13–2.12]; *p* = 0.0066).

We observed limited cGvHD in 269 (12.3%) and extensive cGvHD in 593 (26.9%) patients. No cGvHD was diagnosed in 1117 patients (50.8%), and no data were available for 222 patients (10%).

Cumulative incidence rates of any and extensive cGvHD at 1, 5 and 10 years were 44.7% (95% CI, 42.4–47.1)/30.6% (95% CI, 28.5–32.8), 50.5% (95% CI, 48.0–3.0)/34.5% (95% CI, 32.2–36.8) and 51.1% (95% CI, 48.5–53.6)/34.8% (95% CI, 32.5–37.2) respectively. For univariate regression analysis, see Table S4.

Multivariable analysis indicated that a BMI 25.0 to <30.0 kg/m^2^ was a risk factor for any cGvHD (SHR 1.408 [95% CI, 1.16–1.71]; *p* = 0.0006). Interestingly, mGPS 2 influenced the development positively of any kind and extensive cGvHD (SHR 0.633 [95% CI, 0.46–0.86]; *p* = 0.0034)/(SHR 0.677 [95% CI, 0.48–0.94]; *p* = 0.0250) (for further significant results see Table S4).

## DISCUSSION

To the best of our knowledge, the present study represents the largest prospective dataset of the mGPS, an inflammatory composite score correlating with sarcopenia and cachexia, in patients undergoing alloHCT as a preconditioning biomarker regarding transplantation outcome. In multivariable analyses, we here show the significant impact of the systemic inflammation response measures/biomarkers sALB, CRP and the mGPS on outcome for alloHCT in addition and in comparison to standard alloHCT scores. Nutritional status and inflammation measures are currently not included in established scores predicting outcome, except for the HCT‐CI score, in which obesity but not underweight is assigned one point together with its implication on outcome. Our multivariable analysis data show that an elevated CRP, low sALB or their combination at admission are significant risk factors for OS, PFS and NRM after alloHCT and concur with evidence on outcomes in conjunction with various oncological malignancies as demonstrated in the last decade.^8^, ^10^ Furthermore, application of the mGPS will be extended as an on‐treatment measure for patients' outcome under immunotherapy.^13^, ^28^ In contrast, our study reveals that the familiar BMI is not a statistically important risk factor; neither in the case of malnutrition (<18.5 kg/m^2^) as described by LeBlanc et al.^5^ or recently Doney et al.^6^ nor in obesity/overweight (≥35 kg/m^2^), the latter reported by Sorror et al.^1^ To date, only individual reports assessing small patient cohorts have been published applying the GPS/mGPS to predict outcome in haematological diseases.^16^, ^17^ The highest patient numbers are found in recently published data on autologous HCT with 224 patients.^17^ Their results concur with ours. A study on alloHCT, again at a single institution, has also demonstrated an association between increased inflammation measures and a poorer outcome but in a small number of patients.^29^ Thus, our data rely on the largest cohorts with haematological malignancies, especially with the context of alloHCT and for the first time compared to standard HCT scores.

Besides the CRP/sALB combination, low sALB itself has been described as an important inflammatory biomarker for outcome in adult patients with MDS,^30^ AML,^31^ in AML patients scheduled for alloHCT,^21^ before alloHCT^22^ and in patients undergoing immunotherapy.^32^

Pathophysiologically, inflammation increases capillary permeability and the escape of albumin; albumin's half‐life is also reduced. Hypoalbuminaemia results from and reflects the inflammatory state.^33^ Albumin substitution therefore does not improve the systemic inflammatory response (SIR).^33^

More than 30% of our patients died of NRM; half of them from fungal or bacterial infections, which have not been obviously detectable at admission. Again, low sALB and high CRP at admission are independently associated with these causes of death. This evidence reflects a compromised nutritional and immunological status resulting in a higher risk of infection, an inability to clear these despite active treatment or wound‐healing complications in oncologic patients under treatment.^3^ Malnutrition is associated with less IgA secretion, reduced macrophagic phagocytosis and a weaker lymphocytic mitogen response and complement activation^34^, ^35^ weakening the patient's response to pathogens. Additionally, granulocytopenia, high‐dose immunosuppression and conditioning therapy‐associated mucositis exacerbate the systemic and tissue impairment in patients during alloHCT.

The impact of low sALB and increased CRP on outcome was also observed in the nine patients whom we assessed at admission, but who died before receiving the graft: infections were their main cause of death, and they nearly all revealed low sALB, elevated CRP and a high mGPS.

As mentioned, the main reason for NRM was early infections. Comparing mGPS, HCT‐CI and EBMT scores to predict fatal outcomes due to infections, we note that a high mGPS or HCT‐CI score predicts more accurately than the EBMT score. The reason for this is that these measures take into account the patient's health beyond just transplant‐specific considerations. Interestingly, the higher the mGPS, the earlier the patients die of infections, especially before day +100 with mGPS 2.

The association between low sALB and the occurrence of infections has been known for decades.^36^ Hypoalbuminaemia at 3 months after alloHCT is described as a risk factor for worse NRM, especially due to infections.^37^ These findings are in line with the increased risk for postoperative infections in patients with low albumin.^38^

Up to now, the mechanisms have not been thoroughly elucidated. Elderly people with chronic inflammation have revealed weaker adaptive and innate immune responses,^39^ as a result of changes in mitochondrial metabolism necessary for the immune response.^40^ The recent discussion about inhibiting mitogen‐activated protein kinase or cyclooxygenase 2 (COX2) to boost immunity in older patients^41^ highlights inflammation's influence on immunity. Moreover, obesity, also known to be associated with chronic inflammation, leads to an impaired B‐cell response.^42^ In alloHCT, particularly in patients early after their transplant, distinguishing between the effects of inflammation and immunosuppression on the immune system poses a significant challenge.

The strength of our data is the large number of consecutively transplanted patients, our complete dataset with very long and complete follow‐up, and the early established SOP‐guided nutrition support^26^ initiated at admission.

However, besides its prospective design, we are aware that our survey has certain limitations. The data are collected over a very long time period in which, for example, the entire supportive therapy and the conditioning regimens have changed. On the other hand, more unrelated donor and haplo‐identical transplantations were performed and the patients' age increased significantly. We have no data on pretreatment, and the results would have been too small scale to analyse each patient by diagnosis, pretreatment or remission. We focus on the outcome of all patients in terms of initial inflammation status. Further limitations are regarding patients' nutritional and functional status. We have no data on nutritional screening, weight loss, body composition parameters, muscle mass or functional performance at admission.

Importantly, despite our patients' careful nutritional care, weight loss could not be prevented during their hospital stay. As we previously demonstrated in 100 patients who lost weight by day +30, they had recovered by day +180.^26^ However, it remains unclear how the patients' nutritional status would have evolved at these time points without our counselling and additive nutrition therapy.

Recent studies addressing inflammation or malnutrition show outcome improvement by early nutrition intervention, especially in patients having a CRP between 10 and 100 mg/L.^43^ Furthermore, a monoclonal antibody (ponsegromab) directed against growth differentiation factor 15 (GDF 15) has been tested. GDF 15 modulates inflammation, and the systemic inflammatory response is a mechanism of cancer cachexia.^44^ The study with ponsegromab has shown promising results to reduce cachexia‐associated symptoms in cancer patients under treatment.^45^ The same positive results are shown in a prospective randomized study with a multimodal treatment in cachexia patients.^46^

However, currently there are no data available supporting the hypothesis that intensive nutrition and/or exercise support preceding a planned alloHCT can reduce the systemic inflammation response and improve outcome. This so‐called pre‐habilitation is an established approach in patients undergoing, for example, visceral cancer surgery.^47^

Still, it is uncertain whether aggressive haematological malignancies give us enough time for such a programme.

## CONCLUSIONS

The present study represents the largest prospective dataset analysing the impact of systemic inflammatory response measured by the mGPS in haematological malignancies. It highlights the significant impact of inflammation markers as risk factors and as predictive of alloHCT outcomes. Multimodal therapies, drugs or targeted therapies influencing inflammation should be evaluated in advance of scheduled alloHCT.

## AUTHOR CONTRIBUTIONS

*Conception and design*: Hartmut Bertz, Jürgen Finke, Donald C McMillan and Robert Zeiser. *Financial support*: Robert Zeiser. *Administrative support*: Hartmut Bertz and Robert Zeiser. *Provision of study materials or patients*: Hartmut Bertz, Claudia Wehr, Jörg Sahlmann, Donald C McMillan, Christina Maas‐Bauer, Jesus Duque‐Alfonso, Ralph Wäsch, Julia Nikolaychuk, Christine Greil, Jann Arends, Jürgen Finke, and Robert Zeiser. *Collection and assembly of data*: Hartmut Bertz, Claudia Wehr, Jörg Sahlmann, Donald C McMillan, Christina Maas‐Bauer, Jesus Duque‐Alfonso, Julia Nikolaychuk, Christine Greil, Jann Arends, Jürgen Finke, and Robert Zeiser. *Data analysis and interpretation*: Jörg Sahlmann, Hartmut Bertz, Jürgen Finke, Donald C McMillan, Jesús Duque‐Alfonso, Jann Arends and Robert Zeiser. *Manuscript writing and final approval*: All authors.

## FUNDING INFORMATION

R.Z. was supported by the EU Project number: 517204983; the European Union: EU Proposal No. European Research council (ERC)‐2022‐ADG Project: 101094168—AlloCure (ERC Advanced grant to R.Z.); ERA‐NET Transcan—PIXEL (to R.Z.); ERA‐NET Transcan—SmartCART (to R.Z.); and Deutsche Forschungsgemeinschaft (DFG, German Research Foundation)—SFB‐1479—Project ID: 441891347 (P01 to R.Z.).

## CONFLICT OF INTEREST STATEMENT

All authors declare no conflict of interest with this manuscript.

## PATIENT'S INFORMED CONSENT

All patients signed informed consent for alloHCT and prospective data collection and were treated according to the Declaration of Helsinki.

## Supporting information


Table S1.
Table S2.
Table S3.
Table S4.
Table S5.

Figure S1.
Figure S2.
Figure S3.

## Data Availability

Data are available upon request. Contact: robert.zeiser@uniklinik-freiburg.de.
