# Supplementary material for: The importance of systemic inflammatory response measurements as pretransplant risk factors for outcome after allogeneic haematopoietic cell transplantation
Source: Br J Haematol. 2025 Jul 29;207(4):1517–28. doi: 10.1111/bjh.70049 (PMC12512061; doi:10.1111/bjh.70049)
Supplement: Supplementary file 1 — Table S1. Table S2. Table S3. Table S4. Table S5. [file BJH-207-1517-s002.zip › R01 Supplement Data Tables S1 -S2 @ BJH.docx]

**Table S1** Scores / measures of the patients not included

| modified GPS | N= |
| --- | --- |
| 0 | 2 |
| 1 | 1 |
| 2 | 6 |
| HCT-CI |  |
| 0-2 | 3 |
| ≥3 | 6 |
|  |  |
| median | range |
| BMI (kg/m²) 26.7 | 18.6-40.2 |
| Albumin (g/L) 33 | 26-46 |
| CRP (mg/L) 76 | 3-150 |
| HCT- CI 4 | 1-7 |
| mGPS 2 | 0-2 |

**Abbreviations:** GPS, Glasgow prognostic score; HCT-CI, hematopoietic cell transplantation specific comorbidity Index; BMI, body mass index; CRP, C-reactive protein

**Table S2** Univariate analysis of variables for outcome

|  | **Univariate analysis** | | |
| --- | --- | --- | --- |
| **Variable** | **SHR*** | **95% CI** | **p-Value** |
|  |  |  |  |
| **OVERALL Survival** |  |  |  |
| remission >CR/CP vs. CR1/CP1 | 2.15 | 1.87-2.48 | <0.001 |
| CR>1/CP>1 vs. CR1/CP1 | 1.59 | 1.24-2.06 | 0.0003 |
| mGPS 1 vs 0 | 1.47 | 1.30-1.67 | <0.001 |
| mGPS 2 vs 0 | 2.39 | 2.00-2.86 | <0.001 |
| HCT-CI 1/2 vs. 0 | 1.40 | 1.21-1.63 | <0.001 |
| HCT-CI ≥3 vs. 0 | 1.63 | 1.41-1.87 | <0.001 |
| EBMT score 3-7 vs. 0-2 | 2.29 | 1.85-2.84 | <0.001 |
| CRP >10 mg/L vs. ≤ 10 mg/L | 1.70 | 1.52-1.90 | <0.001 |
| sALB < 35 g/L vs. ≥ 35 g/L | 1.91 | 1.66-2.20 | <0.001 |
| conditioning RIC vs. MAC | 1.39 | 1.23-1.58 | <0.001 |
| BMI <18.5 vs. 18.5-25.0 | 1.340 | 0.95-1.88 | 0.090 |
| BMI 25.0-<30.0 vs. 18.5-25.0 | 1.043 | 0.93-1.18 | 0.49 |
| BMI 30.0-<35.0 vs. 18.5-25.0 | 1.027 | 0.86-1.22 | 0.76 |
| BMI ≥35.0 vs. 18.5-25.0 | 0.947 | 0.67-1.34 | 0.76 |
| Graft PBSC vs. BM | 1.233 | 0.99-1.53 | 0.059 |
| Graft Cord blood vs. BM | 3.545 | 2.83-4.43 | <0.001 |
| Graft BM + PBSC vs. BM | 6.360 | 1.89-21.36 | 0.0028 |
| Graft PBSC cleaned vs. BM | 1.743 | 0.92-3.29 | 0.087 |
| Graft PBSC combined vs. BM | 1.243 | 1.00-1.54 | 0.050 |
| Donor unrelated vs. related | 1.106 | 0.98-1.24 | 0.089 |
| female donor / male recipient | 1.179 | 1.04-1.33 | 0.0083 |
| CMV reactivation risk yes vs. no | 1.048 | 0.94-1.17 | 0.40 |
| aGvHD °I vs. 0 | 0.784 | 0.68-0.90 | 0.0008 |
| aGvHD °II vs. 0 | 0.843 | 0.72-0.99 | .0340 |
| aGvHD °III vs. 0 | 1.140 | 0.96-1.36 | 0.14 |
| aGvHD °IV vs. 0 | 2.661 | 2.12-3.34 | <0.001 |
| aGvHD °II-°IV vs no | 1.21 | 1.08-1.36 | 0.001 |
| aGvHD °III-°IV vs no | 1.62 | 1.41-1.86 | <0.001 |
| aGvHD °III-°IV vs °I | 1.88 | 1.58-2.22 | <0.001 |
| cGvHD limited | 0.47 | 0.40-0.57 | <0.001 |
| cGvHD extensive | 0.53 | 0.47-0.60 | <0.001 |
|  |  |  |  |
| **PROGRESSION-Free Survival** |  |  |  |
| remission >CR/CP vs. CR1/CP1 | 2.091 | 1.84-2.38 | <0.001 |
| CR>1/CP>1 vs. CR1/CP1 | 1.560 | 1.22-2.00 | 0.004 |
| CRP >10 mg/L vs. ≤ 10 mg/L | 1.548 | 1.39-1.72 | <0.001 |
| sALB < 35 g/L vs. ≥ 35 g/L | 1.726 | 1.50-1.98 | <0.001 |
| mGPS 1 vs. 0 | 1.36 | 1.20-1.53 | <0.001 |
| mGPS 2 vs. 0 | 2.11 | 1.81-2.51 | <0.001 |
| HCT-CI 1/2 vs. 0 | 1.227 | 1.07-1.41 | 0.0041 |
| HCT-CI >=3 vs. 0 | 1.396 | 1.22-1.59 | <0.001 |
| EBMT score 3-7 vs. 0-2 | 2.097 | 1.72-2.55 | <0.001 |
| BMI <18.5 vs. 18.5-25.0 | 1.231 | 0.88-1.72 | 0.22 |
| BMI 25.0-<30.0 vs. 18.5-25.0 | 1.032 | 0.92-1.16 | 0.59 |
| BMI 30.0-<35.0 vs. 18.5-25.0 | 0.993 | 0.84-1.17 | 0.93 |
| BMI ≥35.0 vs. 18.5-25.0 | 0.812 | 0.58-1.14 | 0.23 |
| Conditioning RIC vs. MAC | 1.284 | 1.14-1.44 | <0.001 |
| Graft PBSC vs. BM | 1.145 | 0.94-1.40 | 0.19 |
| Graft Cord blood vs. BM | 3.838 | 3.12-4.73 | <0.001 |
| Graft BM + PBSC vs. BM | 4.305 | 1.27-14.54 | 0.019 |
| Graft PBSC cleaned vs. BM | 1.453 | 0.78-2.71 | 0.24 |
| Graft PBSC combined vs. BM | 1.151 | 0.94-1.41 | 0.17 |
| Donor unrelated vs. related | 1.033 | 0.92-1.16 | 0.56 |
| female donor/male recipient | 1.113 | 0.99-1.25 | 0.076 |
| CMV reactivation risk yes vs. no | 0.992 | 0.89-1.10 | 0.88 |
| aGvHD °I vs. 0 | 0.771 | 0.67-0.88 | 0.0002 |
| aGvHD °II vs. 0 | 0.789 | 0.68-0.92 | 0.0019 |
| aGvHD °III vs. 0 | 0.986 | 0.84-1.16 | 0.87 |
| aGvHD °IV vs. 0 | 2.044 | 1.67-2.50 | <0.001 |
| aGvHD °II-°IV vs no | 1.077 | 0.97-1.20 | 0.17 |
| aGvHD °III-°IV vs °I | 1.591 | 1.36-1.87 | <0.001 |
| aGvHD °III-°IV vs no | 1.373 | 1.21-1.56 | <0.001 |
| cGvHD limited vs. none | 0.467 | 0.40-0.55 | <0.001 |
| cGvHD extensive vs. none | 0.484 | 0.43-0.54 | <0.001 |
|  |  |  |  |
| **RELAPSE Incidence** |  |  |  |
| remission >CR/CP vs. CR1/CP1 | 1.776 | 1.49-2.12 | <0.001 |
| >CR1 & CP1 vs. CR1 & CP1 | 1.443 | 1.03-2.03 | 0.035 |
| CRP >10 mg/L vs. ≤ 10 mg/L | 1.178 | 1.02-1.36 | 0.023 |
| sALB < 35 g/L vs. ≥ 35 g/L | 1.046 | 0.87-1.26 | 0.64 |
| mGPS 1 vs. 0 | 1.202 | 1.03-1.41 | 0.021 |
| mGPS 2 vs. 0 | 1.125 | 0.89-1.42 | 0.32 |
| HCT-CI 1/2 vs. 0 | 1.020 | 0.86-1.22 | 0.82 |
| HCT-CI >=3 vs. 0 | 0.916 | 0.77-1.08 | 0.31 |
| EBMT score 3-7 vs. 0-2 | 1.308 | 1.03-1.65 | 0.025 |
| BMI <18.5 vs. 18.5-25.0 | 1.034 | 0.68-1.58 | 0.88 |
| BMI 25.0-<30.0 vs. 18.5-25.0 | 1.026 | 0.88-1.19 | 0.74 |
| BMI 30.0-<35.0 vs. 18.5-25.0 | 0.881 | 0.71-1.10 | 0.26 |
| BMI ≥35.0 vs. 18.5-25.0 | 0.742 | 0.46-1.20 | 0.22 |
| Conditioning RIC vs. MAC | 0.865 | 0.75-1.00 | 0.047 |
| Graft PBSC vs. BM | 1.138 | 0.88-1.46 | 0.32 |
| Graft Cord blood vs. BM | 7.538 | 5.83-9.74 | <0.001 |
| Graft BM + PBSC vs. BM | 0.001 | 0.00-0.00 | <0.001 |
| Graft PBSC cleaned vs. BM | 0.596 | 0.24-1.49 | 0.27 |
| Graft PBSC combined vs. BM | 1.130 | 0.88-1.45 | 0.34 |
| Donor unrelated vs. related | 1.053 | 0.90-1.24 | 0.53 |
| female donor/male recipient | 0.791 | 0.69-0.91 | 0.0013 |
| CMV reactivation risk yes vs. no | 0.898 | 0.78-1.03 | 0.13 |
| aGvHD °I vs. 0 | 0.958 | 0.81-1.13 | 0.62 |
| aGvHD °II vs. 0 | 0.840 | 0.69-1.02 | 0.085 |
| aGvHD °III vs. 0 | 0.755 | 0.59-0.96 | 0.022 |
| aGvHD °IV vs. 0 | 0.371 | 0.24-0.59 | <0.001 |
| aGvHD °II-°IV vs no | 0.737 | 0.63-0.86 | 0.001 |
| aGvHD °III-°IV vs no | 0.654 | 0.53-0.81 | <0.001 |
| aGvHD °III-°IV vs °I | 0.655 | 0.51-0.83 | <0.001 |
| cGvHD limited vs. none | 0.750 | 0.62-0.91 | 0.0041 |
| cGvHD extensive vs. none | 0.449 | 0.38-0.53 | <0.001 |
|  |  |  |  |
| **Non-Relapse-Mortality** |  |  |  |
| remission >CR/CP vs. CR1/CP1 | 1.502 | 1.26-1.80 | <0.001 |
| >CR1 & CP1 vs. CR1 & CP1 | 1.304 | 0.93-1.83 | 0.13 |
| CRP >10 mg/L vs. ≤ 10 mg/L | 1.425 | 1.23-1.65 | <0.001 |
| sALB < 35 g/L vs. ≥ 35 g/L | 1.753 | 1.47-2.09 | <0.001 |
| mGPS 1 vs. 0 | 1.215 | 1.02-1.44 | 0.027 |
| mGPS 2 vs. 0 | 1.964 | 1.58-2.44 | <0.001 |
| HCT-CI 1/2 vs. 0 | 1.281 | 1.05-1.57 | 0.016 |
| HCT-CI >=3 vs. 0 | 1.642 | 1.36-1.98 | <0.001 |
| EBMT score 3-7 vs. 0-2 | 2.207 | 1.64-2.98 | <0.001 |
| BMI <18.5 vs. 18.5-25.0 | 1.214 | 0.79-1.87 | 0.38 |
| BMI 25.0-<30.0 vs. 18.5-25.0 | 1.050 | 0.89-1.23 | 0.55 |
| BMI 30.0-<35.0 vs. 18.5-25.0 | 1.164 | 0.93-1.46 | 0.18 |
| BMI ≥35.0 vs. 18.5-25.0 | 0.991 | 0.62-1.57 | 0.97 |
| Conditioning RIC vs. MAC | 1.700 | 1.44-2.01 | <0.001 |
| Graft PBSC vs. BM | 0.998 | 0.77-1.29 | 0.99 |
| Graft Cord blood vs. BM | 0.002 | 0.00-0.01 | <0.001 |
| Graft BM + PBSC vs. BM | 10.370 | 3.82-28.14 | <0.001 |
| Graft PBSC cleaned vs. BM | 2.117 | 1.07-4.19 | 0.031 |
| Graft PBSC combined vs. BM | 1.012 | 0.78-1.31 | 0.93 |
| Donor unrelated vs. related | 1.290 | 1.10-1.51 | 0.0017 |
| female donor/male recipient | 1.156 | 0.98-1.36 | 0.0850 |
| CMV reactivation risk yes vs. no | 1.184 | 1.02-1.37 | 0.0220 |
| aGvHD °I vs. 0 | 0.753 | 0.61-0.92 | 0.0062 |
| aGvHD °II vs. 0 | 0.952 | 0.77-1.18 | 0.65 |
| aGvHD °III vs. 0 | 1.332 | 1.06-1.67 | 0.013 |
| aGvHD °IV vs. 0 | 4.068 | 3.18-5.21 | <0.001 |
| aGvHD °II-°IV vs no | 1.549 | 1.34-1.79 | <0.001 |
| aGvHD °III-°IV vs no | 2.132 | 1.80-2.52 | <0.001 |
| aGvHD °III-°IV vs °I | 2.616 | 2.09-3.27 | <0.001 |
| cGvHD limited vs. none | 0.548 | 0.43-0.70 | <0.001 |
| cGvHD extensive vs. none | 1.003 | 0.86-1.17 | 0.96 |

**Abbreviations:** CR, complete remission; CP, chronic phase; mGPS, modified Glasgow prognostic score; HCT-CI, hematopoietic cell transplantation specific comorbidity Index; EBMT, European Society for Blood and Marrow Transplantation; CRP, c-reactive protein; sALB, serum-albumin; RIC, reduced intensity conditioning; MAC, myeloablative conditioning; BMI, body mass Index; PBSC, peripheral blood stem cells; BM, bone marrow; CMV, cytomegaly virus; aGvHD, acute graft-versus-host disease; cGvHD, chronic graft-versus-host disease
