# Supplementary material for: The importance of systemic inflammatory response measurements as pretransplant risk factors for outcome after allogeneic haematopoietic cell transplantation
Source: Br J Haematol. 2025 Jul 29;207(4):1517–28. doi: 10.1111/bjh.70049 (PMC12512061; doi:10.1111/bjh.70049)
Supplement: Supplementary file 1 — Table S1. Table S2. Table S3. Table S4. Table S5. [file BJH-207-1517-s002.zip › R01 Table S5 Time dependent GVHD.docx]

**Table S5** Univariate analysis for OS and PFS according time-dependent a/cGvHD

|  | **Univariate analysis** | | |
| --- | --- | --- | --- |
| **Variable** | **HR*** | **95% CI** | **p-Value** |
|  |  |  |  |
| **OVERALL Survival** |  |  |  |
| aGvHD °I vs. 0 | 1.526 | 1.24-1.88 | <0.001 |
| aGvHD °II vs. 0 | 1.659 | 1.33-2.07 | <0.001 |
| aGvHD °III vs. 0 | 2.369 | 1.88-2.98 | <0.001 |
| aGvHD °IV vs. 0 | 5.974 | 4.62-7.72 | <0.001 |
| aGvHD °III-°IV vs 0 | 2.746 | 2.33-3.23 | <0.001 |
| any aGvHD vs. no | 1.934 | 1.61-2.32 | <0.001 |
| cGvHD limited vs. 0 | 0.836 | 0.68-1.03 | 0.098 |
| cGvHD extensive vs. 0 | 0.956 | 0.81-1.13 | 0.601 |
| any cGvHD vs. no | 0.917 | 0.78-1.07 | 0.283 |
|  |  |  |  |
| **PROGRESSION Free Survival** |  |  |  |
| aGvHD °I vs. 0 | 1.735 | 1.42-2.13 | <0.001 |
| aGvHD °II vs. 0 | 1.756 | 1.41-2.18 | <0.001 |
| aGvHD °III vs. 0 | 2.279 | 1.81-2.87 | <0.001 |
| aGvHD °IV vs. 0 | 5.495 | 4.25-7.10 | <0.001 |
| aGvHD °III-°IV vs 0 | 2.584 | 2.20-3.4 | <0.001 |
| any aGvHD vs. no | 2.046 | 1.71-2.45 | <0.001 |
| cGvHD limited vs. 0 | 1.059 | 0.85-1.32 | 0.602 |
| cGvHD extensive vs. 0 | 1.108 | 0.93-1.32 | 0.254 |
| any cGvHD vs. no | 1.093 | 0.93-1.29 | 0.294 |
|  |  |  |  |

**Abbreviations:** aGvHD, acute graft-versus-host Disease; cGvHD, chronic graft-versus-host
