# Supplementary material for: The importance of systemic inflammatory response measurements as pretransplant risk factors for outcome after allogeneic haematopoietic cell transplantation
Source: Br J Haematol. 2025 Jul 29;207(4):1517–28. doi: 10.1111/bjh.70049 (PMC12512061; doi:10.1111/bjh.70049)
Supplement: Supplementary file 1 — Table S1. Table S2. Table S3. Table S4. Table S5. [file BJH-207-1517-s002.zip › Supplement Data Tables S3 -S4 @ BJH.docx]

**Table S3** Time frame of NRM due to infections and scores

| Score |  | N= | Death on median day+ | Range days |
| --- | --- | --- | --- | --- |
|  |  |  |  |  |
| mGPS | 0 | 213 | 238.0 | 1-6695 |
|  | 1 | 99 | 160.0 | 0-2802 |
|  | 2 | 78 | 58.5 | 3-2516 |
|  |  |  |  |  |
| HCT-CI | 0 | 72 | 294.0 | 15-3105 |
|  | 1/2 | 111 | 229.0 | 5-6103 |
|  | ≥3 | 207 | 119.0 | 0-6695 |
|  |  |  |  |  |
| EBMT score | 0/1 | 6 | 433.0 | 162-1927 |
|  | 2 | 12 | 318.0 | 15- 997 |
|  | 3 | 30 | 244.5 | 5-3801 |
|  | ≥4 | 342 | 150.5 | 0-6695 |

**Abbreviations:** mGPS, modified Glasgow prognostic score; HCT-CI, hematopoietic cell transplantation specific comorbidity index; EBMT, European Society for Blood and Marrow Transplantation

**Table S4** Uni- and multivariable risk factors for GVHD

|  | **Univariate analysis** | | | **Multivariable analysis**** | | |
| --- | --- | --- | --- | --- | --- | --- |
| **Variable** | **SHR*** | **95% CI** | **p-Value** | **SHR*** | **95% CI** | **p-Value** |
|  |  |  |  |  |  |  |
| **acute GVHD °II-°IV** |  |  |  |  |  |  |
| not in CR/CP | 1.239 | 1.00-1.54 | 0.0489 |  |  |  |
| PBSC | 1.845 | 1.29-2.70 | 0.0012 | 1.700 | 1.18-2.51 | 0.0060 |
| BMI 30.0-<35.0 vs 18.5-<25.9 | 1.488 | 1.12-1.97 | 0.0057 | 1.469 | 1.10-1.95 | 0.0081 |
| mGPS 1 | 1.223 | 0.99-1.51 | 0.0639 |  |  |  |
| EBMT score 3-7 | 1.418 | 1.04-1.96 | 0.0295 |  |  |  |
| HCT-CI ≥3 | 1.316 | 1.05-1.66 | 0.019 |  |  |  |
|  |  |  |  |  |  |  |
| **acute GVHD °III-°IV** |  |  |  |  |  |  |
| PBSC vs BM | 2.821 | 1.65-5.27 | 0.0004 | 2.522 | 1.46-4.74 | 0.0019 |
| BMI 30.0-<35.0 vs 18.5-25.0 | 1.735 | 1.24-2.40 | 0.0010 | 1.756 | 1.26-2.44 | 0.0009 |
| HCT-CI ≥3 | 1.749 | 1.30-2.38 | 0.0003 | 1.543 | 1.13-2.12 | 0.0066 |
| CRP >10 mg/L | 1.320 | 1.05-1.66 | 0.0172 |  |  |  |
| mGPS 1 | 1.306 | 1.00-1.69 | 0.0442 | 1.283 | 0.98-1.67 | 0.0640 |
|  |  |  |  |  |  |  |
| **chronic GVHD**  **limited or extensive** |  |  |  |  |  |  |
| not in CR/CP | 0.622 | 0.51-0.76 | <0.0001 | 0.682 | 0.53-0.87 | 0.0025 |
| female donor/male recipient | 1.332 | 1.08-1.64 | 0.0063 | 1.258 | 1.01-1.56 | 0.0368 |
| sALB <35g/L | 0.587 | 0.46-0.75 | <0.0001 |  |  |  |
| CRP >10mg/L | 0.753 | 0.63-0.90 | 0.0022 |  |  |  |
| EBMT score 3-7 | 0.630 | 0.48-0.83 | 0.001 | 1.007 | 0.70-1.46 | 0.97 |
| mGPS 2 vs 0 | 0.550 | 0.41-0.74 | <0.001 | 0.633 | 0.46-0.86 | 0.0034 |
| BMI 25.0-<30.0 vs. 18.5-25.0 | 1.408 | 1.16-1.71 | 0.0005 | 1.408 | 1.16-1.71 | 0.0006 |
| BMI ≥35.0 vs. 18.5-25.0 | 1.623 | 0.97-2.70 | 0.0628 | 1.625 | 0.96-2.73 | 0.0662 |
| unrelated donor | 0.657 | 0.55-0.79 | <0.001 | 0.691 | 0.56-0.85 | 0.0003 |
|  |  |  |  |  |  |  |
| **chronic GVHD extensive** |  |  |  |  |  |  |
| female donor/male recipient | 1.382 | 1.11-1.72 | 0.0042 | 1.264 | 1.00-1.59 | 0.0474 |
| not in CR/CP | 0.673 | 0.55-0.83 | 0.0002 | 0.677 | 0.52-0.89 | 0.0042 |
| RIC | 0.784 | 0.64-0.96 | 0.0166 |  |  |  |
| unrelated donor | 0.551 | 0.45-0.67 | <0.001 | 0.554 | 0.45-0.69 | <0.001 |
| mGPS 2 | 0.617 | 0.44-0.85 | 0.0045 | 0.677 | 0.48-0.94 | 0.025 |
| sALB <35g/L | 0.750 | 0.57-0.97 | 0.0324 |  |  |  |
| CRP >10mg/L | 0.766 | 0.63-0.94 | 0.0098 |  |  |  |
| EBMT score 3-7 | 0.695 | 0.52-0.94 | 0.0151 | 1.303 | 0.88-1.93 | 0.18 |

**Abbreviations:** aGvHD, acute graft-versus-host Disease; cGvHD, chronic graft-versus-host Disease; CR, complete remission; CP, chronic phase; PBSC, peripheral blood stem Cells; BMI, body mass index; mGPS, modified Glasgow prognostic score; EBMT, European Society for Blood and Marrow Transplantation; HCT-CI, hematopoietic cell transplantation specific comorbidity index; CRP, c-reactive protein; sALB, serum-albumin
