# Supplementary material for: The importance of systemic inflammatory response measurements as pretransplant risk factors for outcome after allogeneic haematopoietic cell transplantation
Source: Br J Haematol. 2025 Jul 29;207(4):1517–28. doi: 10.1111/bjh.70049 (PMC12512061; doi:10.1111/bjh.70049)
Supplement: Supplementary file 2 — Figure S1. Figure S2. Figure S3. [file BJH-207-1517-s001.zip › R01 Figure S4 Death Relapse vs NRM cum incidences.docx]

**Figure** S4 Cumulative incidences of relapse and non-relapse-mortality


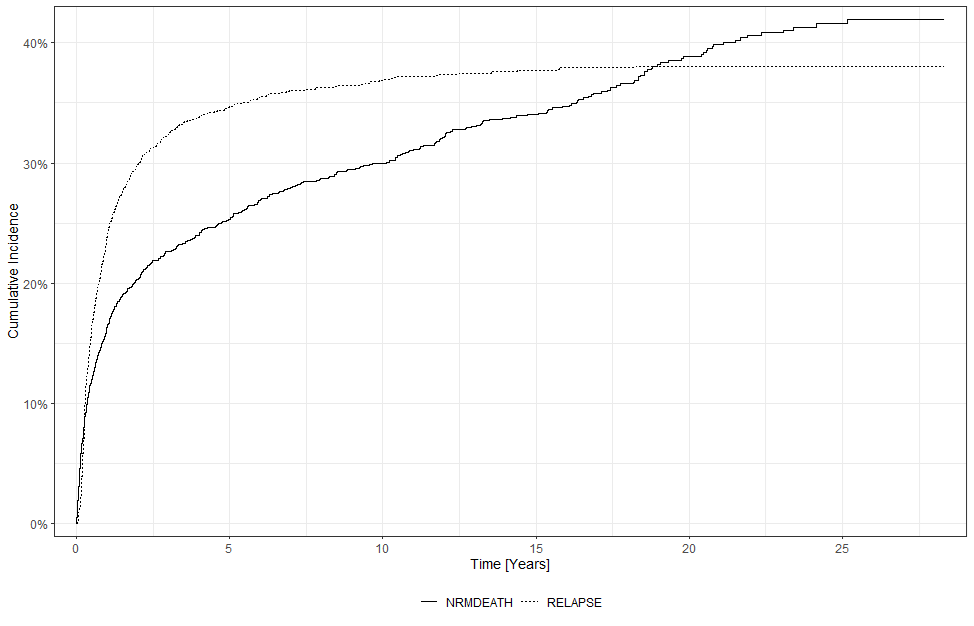


**Abbreviations:** NRM, non-relapse-mortality
