# Supplementary material for: The importance of systemic inflammatory response measurements as pretransplant risk factors for outcome after allogeneic haematopoietic cell transplantation
Source: Br J Haematol. 2025 Jul 29;207(4):1517–28. doi: 10.1111/bjh.70049 (PMC12512061; doi:10.1111/bjh.70049)
Supplement: Supplementary file 2 — Figure S1. Figure S2. Figure S3. [file BJH-207-1517-s001.zip › Supplement data Figures S1 - S 2 @ BJH.docx]

**Figure** S1

(A)


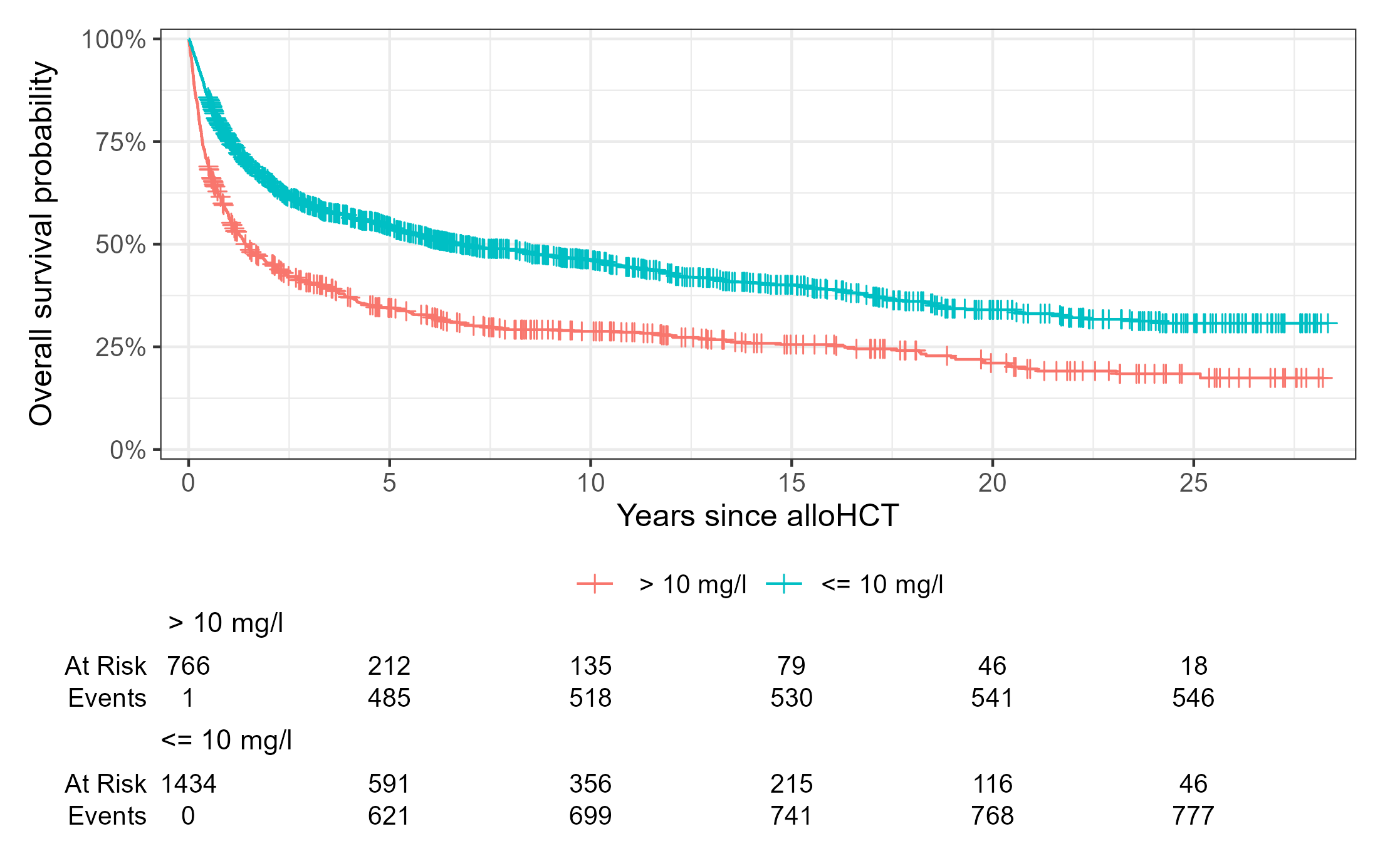


(B)


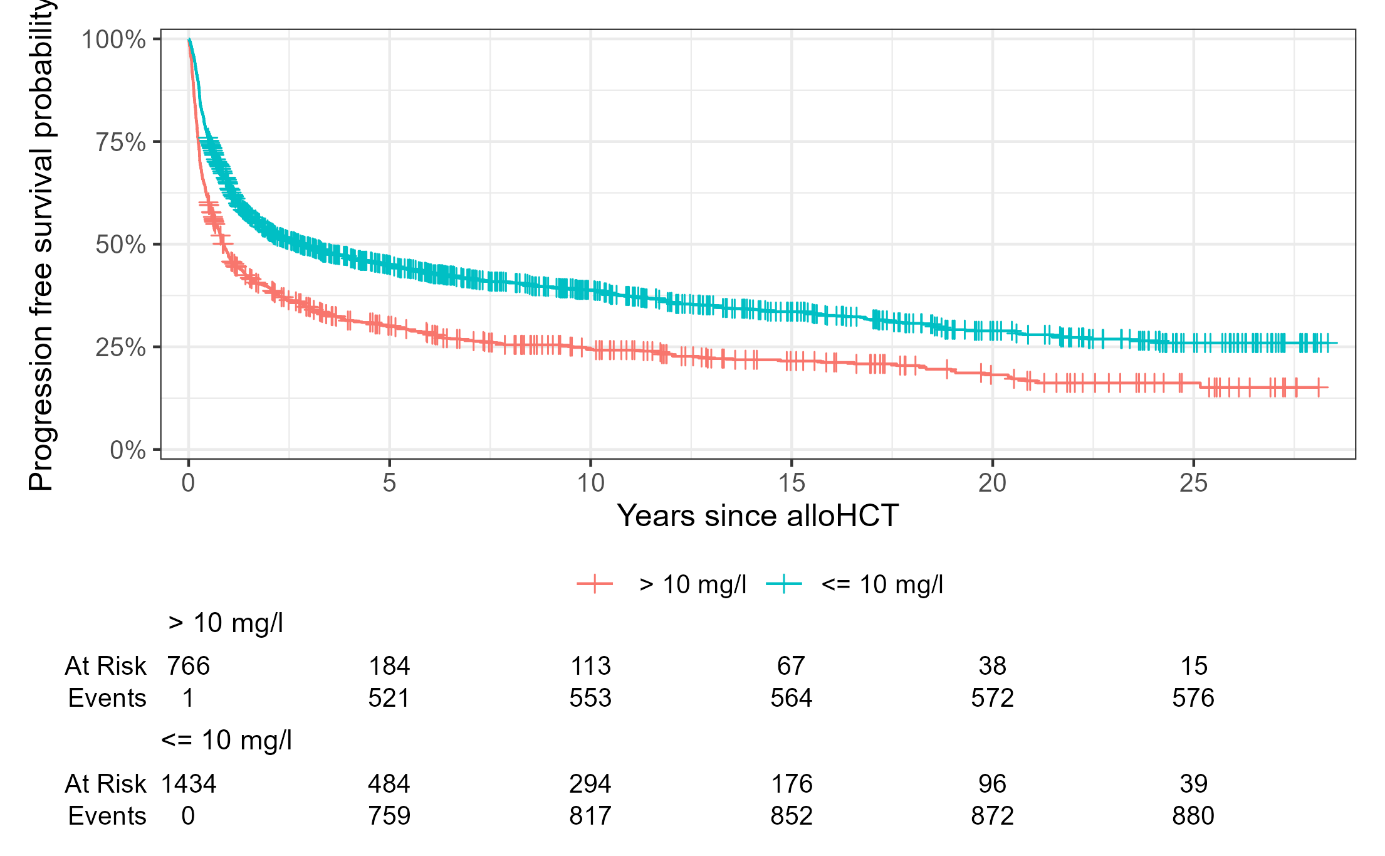


**Figure S1:** Kaplan-Meier curves showing the relationship normal and pathological CRP regarding (A) overall survival (B) progression free survival

**Figure** S2

(A)


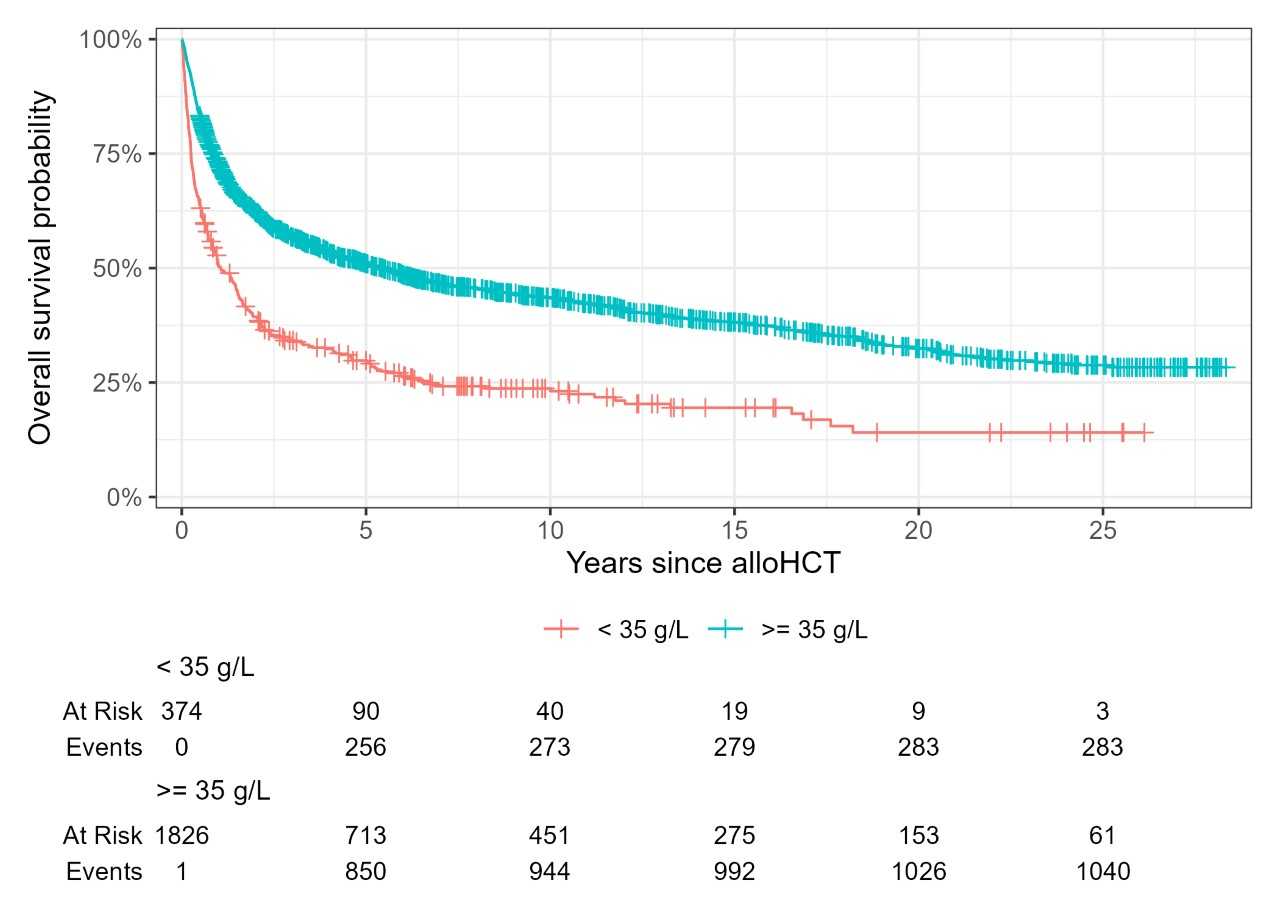


(B)


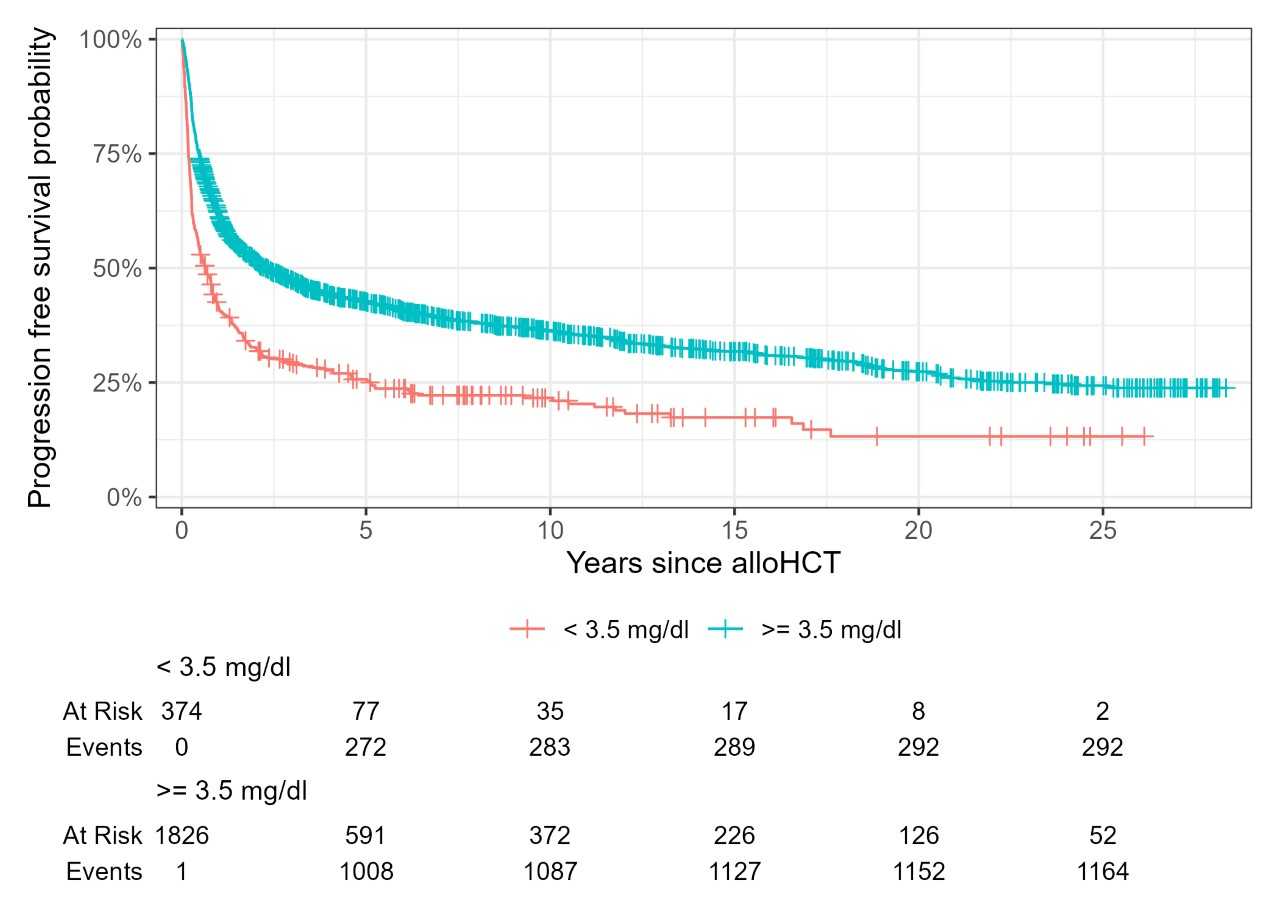


**Figure S2:** Kaplan-Meier curves showing the relationship normal and pathological Serum-Albumin regarding (A) overall survival (B) progression free survival
